# Vector Vortex Beam-Enabled Edge Microscopy with Dynamic Orientation Selectivity

**DOI:** 10.1021/acsphotonics.5c02355

**Published:** 2025-11-25

**Authors:** Hammad Ahmed, Muhammad Afnan Ansari, Lynn Paterson, Xibin Yang, Xianzhong Chen

**Affiliations:** † Institute of Photonics and Quantum Sciences, School of Engineering and Physical Sciences, 3120 Heriot-Watt University, Edinburgh EH14 4AS, U.K.; ‡ Institute of Biological Chemistry, Biophysics and Bioengineering, School of Engineering and Physical Sciences, Heriot-Watt University, Edinburgh EH14 4AS, U.K.; § 681045 Suzhou Institute of Biomedical Engineering and Technology, Chinese Academy of Sciences, Suzhou, Jiangsu 215163, China

**Keywords:** optical metasurfaces, edge imaging, optical vortex beam, vector vortex beam

## Abstract

Edge enhancement is an image processing technique that aids in highlighting structural details, especially in samples with weak scattering properties or low intrinsic contrast. While traditional methods such as scalar vortex-based filtering provide isotropic edge detection, they lack orientation selectivity and often introduce artifacts when modified for anisotropic imaging. Here, we present a multifunctional edge imaging system based on vector vortex beams that can be used for dynamic polarization control for orientation-selective edge enhancement. The system integrates a metasurface (Vector Vortex beam) VVB generator within a 4f Fourier transform configuration, along with a pair of rotating half-wave plates that enable tunable spatial polarization distributions. This configuration allows real-time visualization of edge features aligned along specific orientations. We demonstrate the effectiveness of this approach through experiments on resolution charts and label-free imaging of *Saccharomyces cerevisiae* yeast cells. The ability to selectively highlight edge features with dynamic control makes our system a powerful tool for high-contrast, orientation-resolved imaging in microscopy, biomedical diagnostics, and optical inspection.

## Graphical abstract

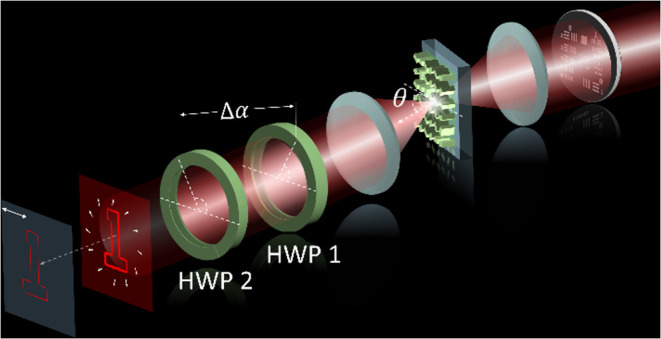

## Introduction

Edge enhancement is a fundamental image processing technique that plays a crucial role in revealing structural details of objects, particularly when they are weakly scattering or embedded in low-contrast environments.^1,2^ It has been extensively utilized across various fields, including image processing,^3^ microscopy,^4,5^ and astronomy.^6^ Traditional techniques, such as spatial filtering in a 4f optical configuration, have long been employed for this purpose.^7^ Radial Hilbert transform (RHT) filtering using a scalar vortex phase mask has proven effective for isotropic edge enhancement due to its characteristic signum-like phase variation around the vortex core.^8,9^ Although the vortex phase is azimuthal, the RHT produces a radially symmetric effect in the spatial domain, enhancing features isotropically in all directions.^9^ These scalar vortex filters have demonstrated effectiveness not only in edge detection but also in enhancing phase contrast in microscopy, making them valuable for imaging transparent samples.

Recently, orientation selectivity has been explored to extend the capabilities of conventional scalar vortex filtering. This has been achieved through approaches such as fractional vortex filters, astigmatic transformations, or shifted spiral phase modulation.^10–12^ However, these techniques inherently modify the radial symmetry of the filtering process, which often leads to unwanted shadow effects and asymmetries in the reconstructed image, hindering coronagraphic imaging and edge detection.^4^

Unlike scalar vortices, vector vortex beams (VVBs) exhibit spatially varying polarization distributions, which can interact with analyzers to reveal polarization-dependent edge features.^13^ These beams can be generated through the superposition of right and left circularly polarized vortex beams.^14^ In recent years, optical metasurfaces have emerged as a compact platform for generating such structured light fields, owing to their unprecedented ability to control the phase, amplitude, and polarization of light at subwavelength scales.^15,16^ This has facilitated the realization of various vortex beams, including perfect vortex beams,^17,18^ composite vortices,^19^ vector beams,^20–22^ and on-chip OAM source.^23,24^ Moreover, metasurfaces have been integrated into a range of imaging systems, where they enhance the capabilities of state-of-the-art techniques, such as polarimetric imaging,^25^ isotropic edge imaging,^26–28^ spectral imaging,^29–31^ and 3D imaging.^32–35^ These developments provide a robust foundation for meta-optics-based imaging systems, enabling the extraction of local edge features aligned along specific orientations while mitigating the limitations associated with traditional scalar filtering methods.

Cotrufo et al. proposed a polarization-based edge detection scheme using a metasurface with a C6 rotational symmetry, which is originally polarization insensitive under normal incidence. However, at higher incident angles, this symmetry breaks, resulting in a Laplacian-like polarization response that enables orientation-sensitive edge detection.^36^ Furthermore, under oblique incidence, it exhibits a Laplacian-like polarization response that becomes sensitive to alignment. This enhanced polarization response under angled illumination makes the system vulnerable to fabrication inaccuracies, as the polarization amplitude is highly sensitive to the geometric parameters of the unit cell. In another approach, Zhou et al. employed a spin-to-orbit interaction-based metasurface spatial differentiator to perform orientation-dependent edge imaging.^37^ Nonetheless, their method relies on a fixed polarization profile, requiring physical reorientation of the metasurface to target edges in different directions, thereby limiting real-time reconfigurability

In this study, we present a multifunctional edge imaging system based on VVBs and dynamic polarization control. The imaging system is designed to selectively enhance object boundaries with directional sensitivity. Our approach utilizes a metasurface-based VVB generator embedded within a 4f Fourier imaging system, along with two rotating half-wave plates (HWPs) placed after the Fourier plane. The relative orientation of these HWPs enables the continuous modulation of the spatial polarization distribution without altering the incident beam or the structural layout of the imaging system. When combined with a linear analyzer, this tunable polarization profile enables dynamically reconfigurable edge contrast, facilitating real-time, orientation-specific visualization of structural features. We evaluate the performance of this system using both resolution targets and biological samples such as *Saccharomyces cerevisiae* (brewer’s yeast) cells for label-free imaging. Our results demonstrate the clear visualization of cellular contours otherwise obscured in conventional bright-field microscopy. The proposed method thus combines the strengths of VVBs and polarization engineering to offer a compact, flexible, and label-free imaging platform, which has potential applications in biomedical optics and material characterization.

## Results

Figure 1 illustrates the schematic of a VVB-based multifunctional microscopy system for selective edge enhancement. The imaging setup is a Fourier transform (FT) configuration, where a metasurface-based VVB generator is placed at the Fourier plane. When an incident light beam illuminates a target, edge images with various orientations are revealed (after passing through an analyzer) based on the polarization profile of the vector vortex beams. Two HWPs are used to further modify the polarization profile of the generated vector vortex beams based on the difference of fast axes, allowing dynamic visualization of local edge features along specific orientations without disturbing the overall imaging system. This approach effectively decouples the image detection plane from the 4f imaging system.

**Figure 1:**
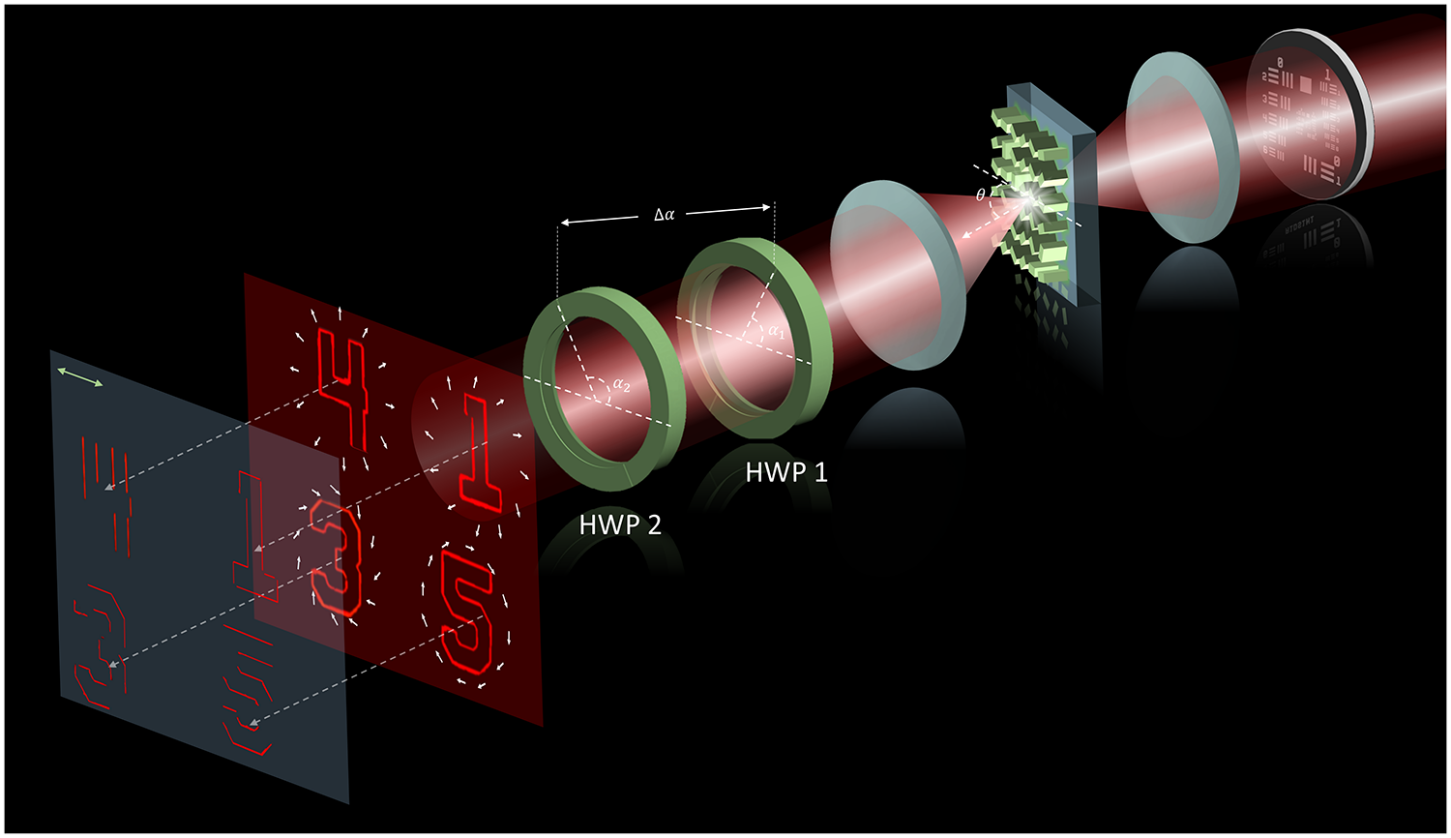
Schematic of a VVB-based multifunctional microscopy system for selective edge enhancement. The setup utilizes a 4f imaging configuration with a metasurface-based VVB generator placed at the Fourier plane. The interaction between the incident light and a sample produces edge-enhanced images at the detection plane, which are dynamically modulated by the polarization-dependent properties of the VVBs. Two HWPs adjust the polarization profile by varying their relative fast-axis orientations, enabling dynamic control over edge directionality without altering the core imaging system.

To design such an imaging system, we start from the VVB generation, which can be represented as the superposition of two optical vortices (OVs) with different circular polarization states.

1 $$E_{\mathrm{VVB}}=E_{R}e^{i\left(\varphi_{o}\right)}\left|R\right\rangle +E_{L}e^{-i\left(\varphi_{o}\right)}\left|L\right\rangle$$

where

$$\left|R\right\rangle =e^{\mathrm{im}\varphi }\left[\begin{array}{l} 1\\ -i \end{array}\right]$$

and

$$\left|L\right\rangle =e^{-\mathrm{im}\varphi }\left[\begin{array}{l} 1\\ i \end{array}\right]$$

are OVs with right circular polarization (RCP) and left circular polarization (LCP), respectively. φ_*o*_ is the initial phase and φ is the azimuthal angle. To generate these beams, we employed a plasmonic metasurface featuring an off-axis configuration. This design avoids the unconverted part. The metasurface is composed of silver (Ag) nanorods, whose orientation angles (ϕ) vary spatially according to a defined distribution.

2 $$\phi =\frac{1}{2}\arg\left(E_{o}e^{i\left(m\varphi +\delta \right)}+E_{o}e^{i\left(m\varphi -\delta \right)}\right)$$

where δ phase difference between neighboring unit cells obtains a phase gradient along the *x*-direction, and *m* is the polarization order. Figure 2a (left) shows the desired phase profile of the metasurface to generate a VVB. Figure 2a (right) shows an SEM image of the fabricated device, showing Ag nanorods with various rotational angles ϕ on an ITO-coated glass substrate. The details about optical performance and nanofabrication are provided in Supporting Sections 1 and 2, respectively. Figure 2b shows the simulated (first row) and experimentally (second row) measured intensity profiles of VVB with *m* = 1 under different linear polarization states (0°, 45°, 135°, and 90°). Blue arrows represent the polarization state of the incident light, whereas white arrows show the polarization distribution of the generated VVB. The analyzer with a transmission axis fixed along the horizontal direction is used to measure the polarization profile (see Supporting Section 3 for more details).

**Figure 2:**
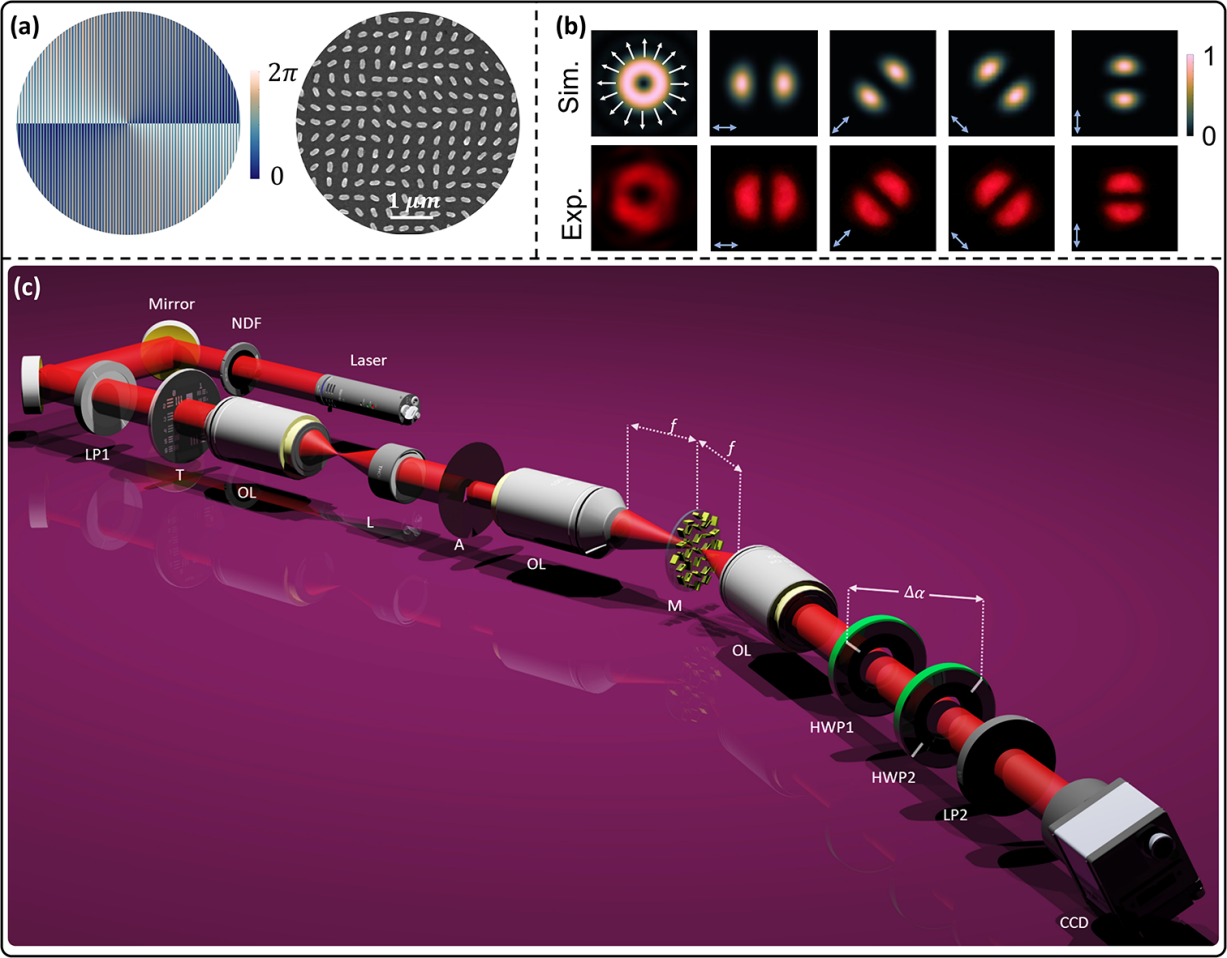
Design, fabrication, and characterization of metasurface devices and a schematic of the imaging system. (a) Left: Designed the phase profile. Right: SEM image of the fabricated sample. (b) Simulated (top row) and measured (bottom row) intensity distributions of generated VVB with *m* = 1 under different linear polarizations. The white arrows indicate the radial polarization profile of the VVB when illuminated with horizontally polarized light. The first column shows the intensity distribution without an analyzer, while the second to fourth columns display the modulated lobe-shaped patterns obtained with different incident polarizations (blue arrows). The transmission axis of the analyzer is fixed along the horizontal direction. (c) Schematic of the experimental setup for imaging. LP1 and LP2 denote linear polarizers; OL1, OL2, and OL3 are 20× objective lenses with a 19 mm working distance; L is a plano-convex lens with a 100 mm focal length; the aperture is rectangular; M represents the metasurface; HWP1 and HWP2 are half-wave plates; NDF is a neutral density filter; T is the Target sample; and CCD refers to the charge-coupled device used for image capture.

Figure 2c shows a schematic of an imaging setup to perform directional edge imaging using a VVB generator. A linearly polarized light beam at an operating wavelength of 650 nm is generated with a supercontinuum laser source (NKT Photonics). The first objective (OL1) with a magnification of 20× and a plano-convex lens (L) with a focal length of 100 mm is used to generate an image of a target (T) on a rectangular aperture. OL2 with a magnification of 20× performs the FT of a target. A VVB generator placed at the Fourier plane modifies the target’s spectrum by spatially variant polarization profiles. OL3 transforms the result from the Fourier plane to an image plane. HWP1 and HWP2 are used to further modify the generated spatial polarization profile based on the difference in angle between the two fast axes (Δα). The final directional edge image can be captured with a CCD camera. A second linear polarizer (LP2) acts as an analyzer and can indirectly confirm the polarization profile and reveal the orientation-selective edge-enhanced images.

We used a commercially available USAF resolution chart to assess the imaging performance. Figure 3 illustrates both the simulated and experimental directional edge imaging of element 3 in group 3. The effects of different polarization distributions (e.g., azimuthal, radial, diagonal, and antidiagonal) on the edges of the target are depicted in Figure 3 (see Supporting Section 4 for imaging of different elements from the target). A quarter-wave plate (QWP) and a linear polarizer (LP) are used to generate the required polarization states. Six polarization states, including RCP, horizontal polarization, 45°, 135°, vertical polarization, and LCP, are selected. These polarization states are geometrically illustrated as points on the Poincaré sphere. The circularly polarized (CP) states produce a simple intensity profile characterized by two concentric rings. It is important to note that no HWPs are used to modify the polarization; only the incident light is altered to achieve the desired polarization profiles. The quantitative analysis is shown in Supporting Section 5.

**Figure 3:**
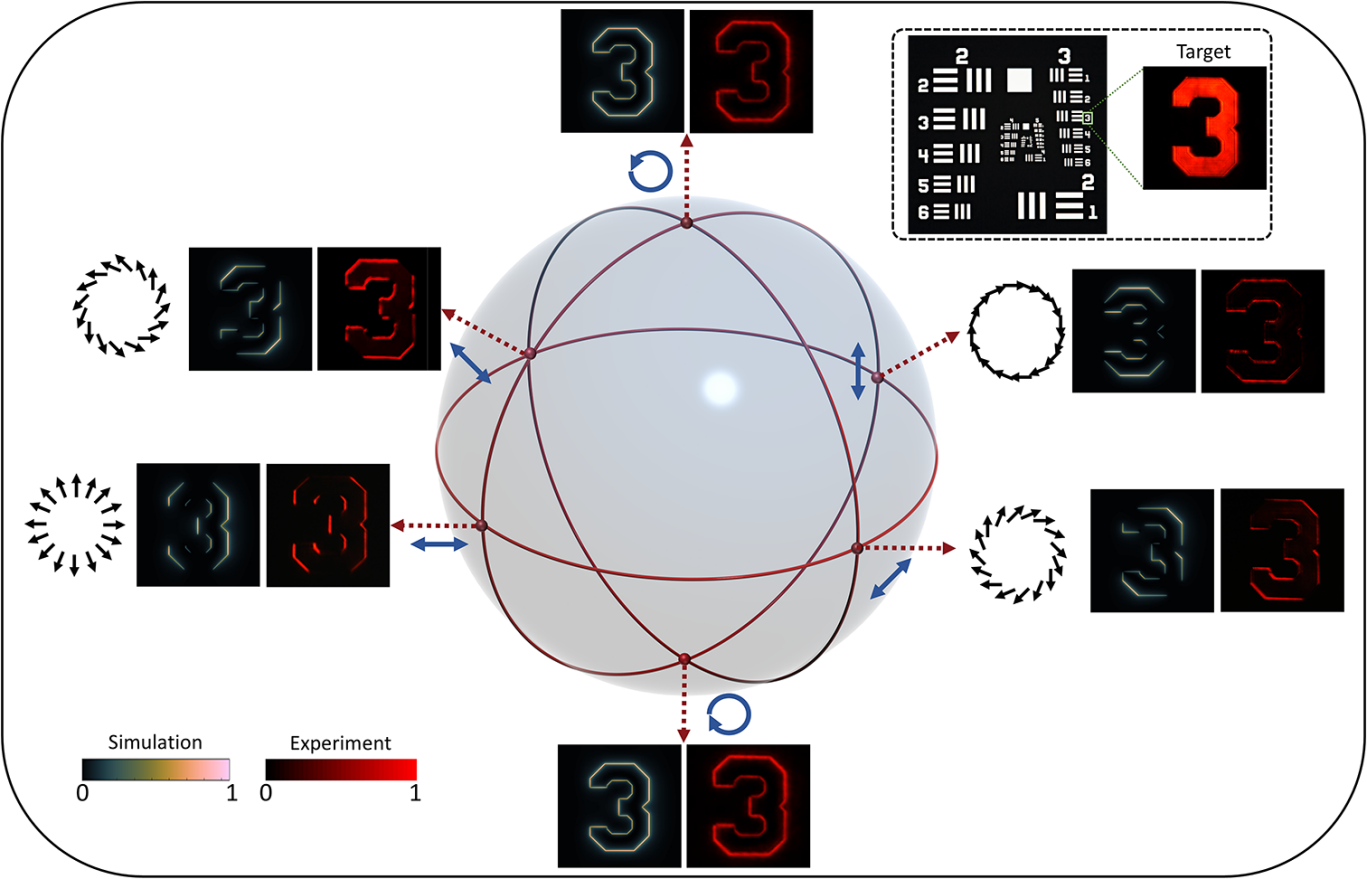
Directional edge imaging with various incident polarization states. Simulation and experimental results are presented for different incident polarization states. A QWP is placed after an LP to generate various polarization states, which are mapped along two different meridians on the Poincaré sphere. Blue arrows represent the incident polarization states. Black arrows illustrate the resulting polarization distributions when the sample is illuminated with linearly polarized light at different orientations. The inset shows the USAF resolution test target, with a captured bright-field image highlighting the number “3”.

To perform dynamic edge imaging, polarization control of the generated VVB is implemented. Two HWPs are inserted between the 4f system and the analyzer. The underlying mechanism is illustrated in Figure 4a. The green arrow indicates the original polarization direction. The angle between this direction and the fast axis (blue arrow) of HWP1 is α_1_. After passing through HWP1, the polarization direction (dashed green arrows) is rotated by 2α_1_. HWP2, whose fast axis is represented by the orange arrow, makes an angle of α_2_ with respect to the original polarization direction. The final polarization, indicated by the pink arrow, is rotated by 2­(α_2_ – α_1_). This rotation depends solely on the relative angle between the fast axes of the two HWPs, providing an additional degree of freedom for dynamic control of the polarization distribution independent of the initial polarization. With the help of Jones calculus, the modified VVB can be expressed as (see Supporting Section 6 for details)

3 $$J_{\mathrm{HWP}}E_{\mathrm{VVB}}=\left[\begin{array}{l} \cos\left(2\left(\alpha_{2}-\alpha_{1}\right)+m\theta +\varphi_{o}\right)\\ \sin\left(2\left(\alpha_{2}-\alpha_{1}\right)+m\theta +\varphi_{o}\right) \end{array}\right]$$

This equation demonstrates that the outgoing beam remains a VVB, with its polarization direction rotated by 2­(α_2_ – α_1_). We apply this principle in our imaging system to obtain edge-enhanced output images as vector fields that are independent of incident polarization. To dynamically visualize local edge features along specific orientations, we select element 4 of group 3. Figure 4b shows the original polarization state of the VVB. The new polarization states, obtained by varying the combinations of fast-axis angles of the HWPs, are shown in the first row of Figure 4c. The corresponding experimental intensity profiles from imaging the USAF target are presented in the second row of Figure 4c. These new polarization profiles are indirectly confirmed when the beam passes through a linear analyzer. To further demonstrate the applicability of the proposed imaging system, we performed experiments on biological samples. Specifically, we image yeast cells in liquid media to highlight local edge features (3rd row of Figure 4c). These cells are prepared on standard microscope slides. Details of sample preparation are provided in the Methods section. Dynamic polarization control enables enhanced visualization of cellular boundaries, demonstrating the potential of this method for biological imaging and label-free contrast enhancement.

**Figure 4:**
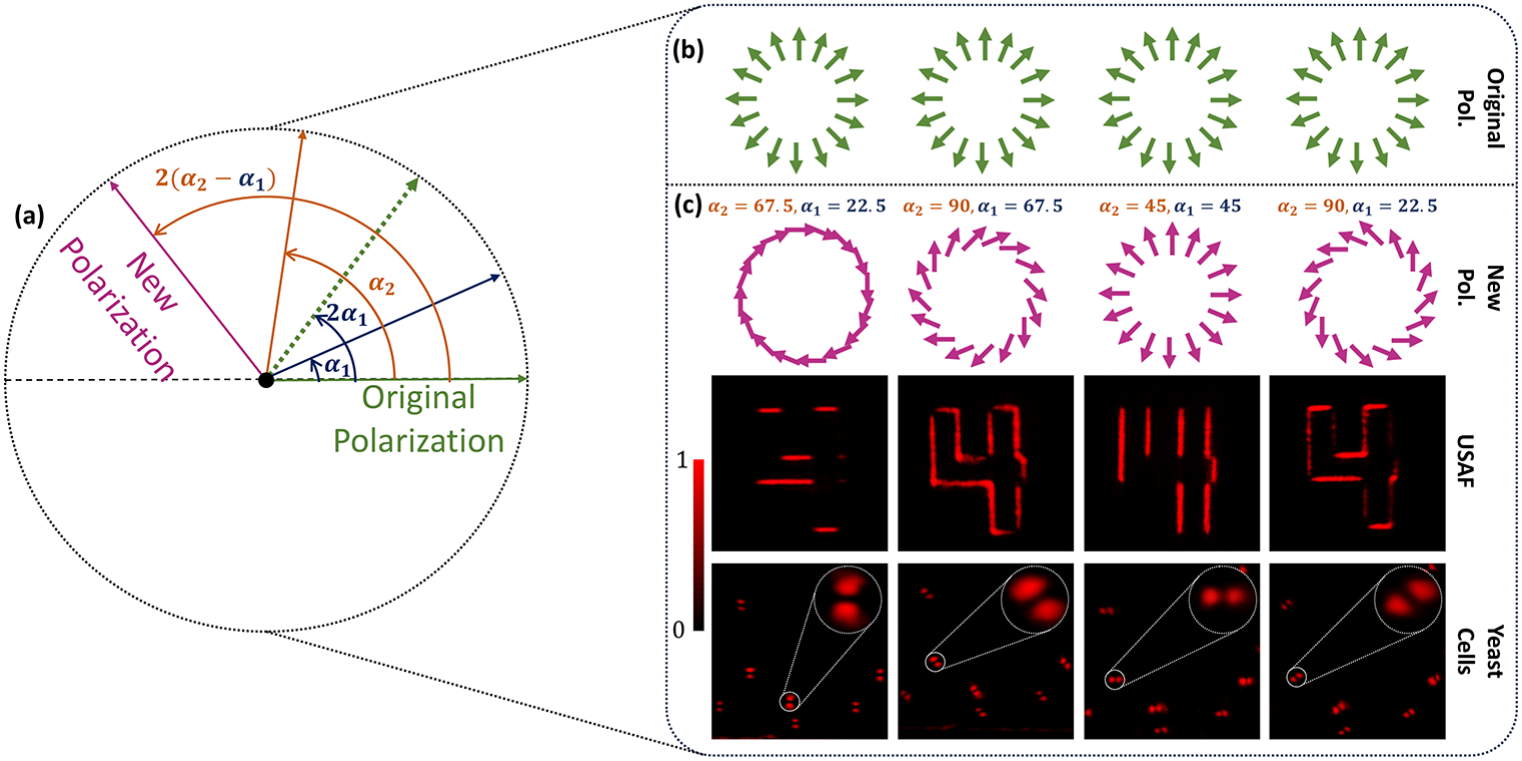
Mechanism of dynamic polarization control and experimental results. (a) Schematic of the dual HWP-based polarization modification. Green and pink arrows represent the original and new polarization states, respectively. Blue and orange arrows are the fast axes of 1st and 2nd HWPs, respectively. α_1_ and α_2_ are the angular positions of fast axes with respect to the original polarizations. The 1st HWP rotates the polarization by 2α_1_, resulting in an intermediate state, represented by the green dashed arrow. The 2nd HWP further rotates this intermediate state by 2­(α_2_ – 2α_1_). The total rotation between the green and pink arrows is 2­(α_2_ – α_1_), which is independent of the initial state of polarization. (b) Original polarization state of VVB. (c) New polarization states based on various combinations of fast axes angles (1st row). Measured intensity profiles of element 4 of group 3 (2nd row) and yeast Cells (3rd row) were obtained after passing through an analyzer.

## Discussion

We have experimentally demonstrated a metasurface-based imaging system capable of revealing orientation-specific edge features of target samples, enabling label-free real-time visualization of structural boundaries. To further demonstrate the imaging capability of the proposed microscopy system, we have conducted additional experiments using biological samples such as Ipomoea root tissue and unstained onion epidermal cells. (Supporting Section 7). The field of view (FOV) was measured with a calibration scale (see Supporting Section 8).^38^ The effective FOV of our imaging system is approximately 0.7 mm, which is sufficient to cover both the resolution chart and biological samples used in our experiments.” The system uses VVBs and polarization control to extract structural features of objects with directional selectivity. This platform is particularly promising for biological imaging, where subtle variations in the refractive index and low inherent contrast often obscure important morphological details. To verify the concept, we employed a plasmonic metasurface composed of Ag nanorods. Although these plasmonic structures offer ease of fabrication and proof-of-concept validation, they inherently suffer from a low conversion efficiency. This limitation can be significantly mitigated by using dielectric metasurfaces, which offer higher efficiency and lower losses in the visible regime.^39^ Additionally, alternative high-efficiency platforms, such as liquid crystal geometric phase optical elements, can achieve over 90% conversion efficiency and offer greater electro-optic tunability.^40,41^

While a 4f Fourier transform setup is used here to validate the concept, the system can be replaced with a large area multifunctional metasurface combining Fourier transformation, vortex generation, and polarization control.^42–44^ This approach can enable a fully lensless, compact, directional edge imaging system.

An additional benefit of our system lies in the independent tunability of the polarization orientation, which is achieved by adjusting the relative angle between the fast axes of two HWPs. A single HWP can rotate the polarization of a linearly polarized beam with the rotation angle depending on the alignment between the polarization direction of the beam and the fast axis of HWP. This works well for beams with a uniform polarization profile. However, when the polarization is spatially variant, as in a VVB with polarization order *m* = 1 (radial polarization), a single HWP rotates different regions of the beam by different amounts, resulting in incorrect polarization states. For example, such a distortion can produce an erroneous *m* = −1 (antivortex radial polarization) (see Figure S6 in Supporting Section 6). To address this issue, we used two HWPs placed in series, with their optical axes oriented at a specific angle relative to each other. This configuration uniformly rotates all polarization vectors regardless of their spatial variation in the incident beam. Unlike previous approaches, our design allows for real-time control of the polarization distribution without modifying the incident beam or altering the imaging setup. This not only improves the practicality and versatility of the imaging platform but also enhances its robustness to fabrication imperfections, as it decouples the polarization response from the angular sensitivity and geometric deviations of the metasurface.

## Conclusion

In summary, we demonstrated a VVB-based multifunctional microscopy system capable of orientation-selective edge enhancement through dynamic polarization control. By manipulating the polarization profiles of VVBs using two rotatable HWPs, we achieve tunable edge visualization without altering the incident beam or modifying the 4f imaging setup. This unique configuration enables decoupling of the image detection plane from the Fourier processing plane, thus enhancing system flexibility and robustness.

## Methods

### Yeast Cells Preparation

Dried, fast-action yeast (*S. cerevisiae*) cells were suspended in Milli-Q water containing 5% YPD (yeast extract, peptone, dextrose) and left to rehydrate for 1 h at 20 °C. Sample chambers were made by applying vinyl stickers to glass microscope slides to form 80 μm deep wells in which 20 μL of the sample was pipetted. Glass coverslips were placed on top. Samples were mounted vertically in the imaging system.

## Supplementary Material





## Data Availability

The data that support the findings of this study are available from the corresponding author upon request.

## Notes

Optical performance, fabrication details, VVB charaterization, directional edge imaging for different USAF chart targets, quantitative analysis, VVB polarization modification, and correction, directional edge imaging of Ipomoea root and onion epidermal cell, field of view measurement, motorized control (PDF)
Motorized Control (Video) (MP4)
