# Supplementary material for: Vector Vortex Beam-Enabled Edge Microscopy with Dynamic Orientation Selectivity
Source: ACS Photonics. 2025 Nov 25;12(12):7013–9. doi: 10.1021/acsphotonics.5c02355 (PMC12715833; doi:10.1021/acsphotonics.5c02355)
Supplement: Supplementary file 1 [file ph5c02355_si_001.pdf]

Supplementary Information

Vector Vortex Beam Enabled Edge Microscopy with Dynamic Orientation Selectivity

*Hammad Ahmed[1], Muhammad Afnan Ansari[1], Lynn Paterson[2], Xibin Yang[3] and Xianzhong Chen[1,]**

[1]Institute of Photonics and Quantum Sciences, School of Engineering and Physical Sciences, Heriot-Watt University, Edinburgh EH14 4AS, UK

[2]Institute of Biological Chemistry, Biophysics and Bioengineering, School of Engineering and Physical Sciences, Heriot-Watt University, Edinburgh, EH14 4AS, UK

[3]Suzhou Institute of Biomedical Engineering and Technology, Chinese Academy of Sciences, Suzhou, Jiangsu 215163, China

Supplementary Section 1: Optical Performance

The schematic of the designed meta-atom (nanorod) is provided in the inset of **Figure S1**. Each nanorod has geometric parameters of length L=200 nm, width W=80 nm, and height H=40nm, with a pixel period of P=300 nm along both the x- and y-directions. **Figure S1** presents the simulated conversion efficiency, obtained using the frequency-domain solver in CST Microwave Studio. In the simulation, the silver permittivity follows the Drude model, while the glass substrate has a refractive index of 1.46. Periodic boundary conditions are applied in the x- and y-directions, and an open boundary condition is set along the z-direction. The results indicate that the conversion efficiency is relatively uniform across the target wavelength range of 500–700 nm.

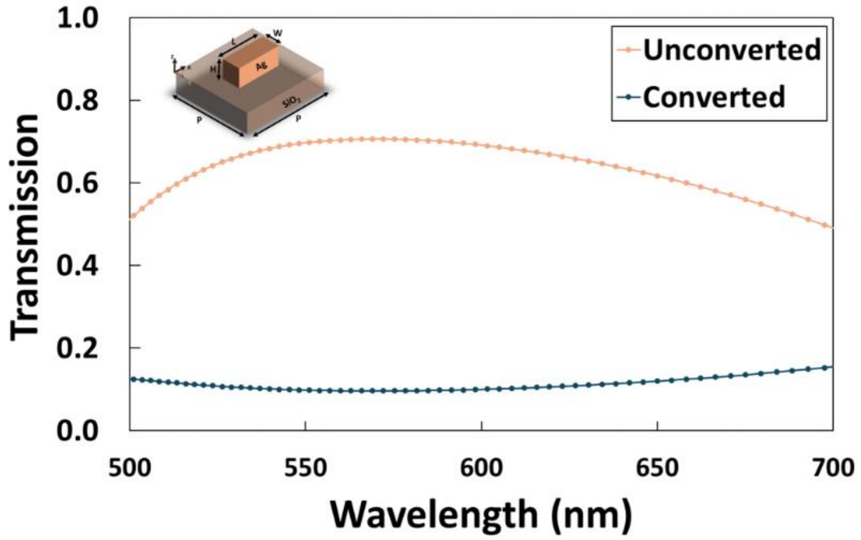

Figure S1. Optical performance analysis of Ag nanorods. Converted and unconverted transmission efficiency. The inset illustrates the schematic of Ag nanorod sitting on a glass substrate.

Supplementary Section 2: Fabrication Details

The proposed metasurface is composed of silver nanorods with spatially varying orientations, fabricated on ITO-coated glass substrates. The fabrication process begins with substrate cleaning, where the glass is sonicated in acetone for 10 minutes, followed by isopropyl alcohol (IPA) for another 10 minutes. A layer of poly (methyl methacrylate) (PMMA) 950 A2 resist is

then applied by spin-coating at 1000 rpm for 60 seconds, resulting in a film approximately 100 nm thick. The coated substrates are soft baked on a hotplate at 180 °C for 5 minutes to improve resist adhesion. Next, electron beam lithography (Raith PIONEER system, 30 kV) is employed to define the nanorod patterns. The exposed samples are developed in a solution of MIBK:IPA (1:3 ratio) for 45 seconds and subsequently rinsed in IPA for 45 seconds. A 40 nm silver layer is deposited using electron beam evaporation. Finally, the lift-off process is performed to remove the unwanted resist and metal, leaving behind the patterned metasurfaces, which are then ready for optical characterization.

Supplementary Section 3: VVB Charaterization

A Supercontinuum laser (NKT Photonics SuperK EXTREME) is used as the light source to characterize the performance of the metasurface device. In our experiment, the wavelength of is 650 nm is used. A linear polarizer and a quarter wave plate (QWP) are used to control the polarization state of the incident light. The generated OV beam intensity profile is displayed on the screen. An iPhone camera is used to capture the screen. A schematic of experimental setup is shown in **Figure S2**. Upon illumination of RCP light, a pair of off-axis centrosymmetric OV beams with $l = +1$ are simultaneously generated (as illustrated in **Figure S2 (a)**). By switching the helicity of incident light from RCP to LCP, the propagating directions of two generated OV beams are swapped with respect to the axis of incident light. Meanwhile, the sign of topological charge (TC) is flipped, i.e., $l = -1$, since the sign of the abrupt phase change introduced by the metasurface is dependent on the helicity of circular polarization (**Figure S2 (b)**) [1]. When a linearly polarized light impinges onto the metasurface, the superimposed OV beam with $\ell = 1$ and $\ell = -1$ is generated. The superimposed beam can be diagnosed (in the form of lobes) by passing the resultant beam through an analyzer (**Figure S2(c)**) and forming an angle α with respect to the horizontal axis. The Jones matrix of an analyzer can be given as [2]

$$\boldsymbol{A} = \begin{bmatrix} \cos^2\alpha & cos\alpha sin\alpha \\ cos\alpha sin\alpha & sin^2\alpha \end{bmatrix}$$

The transmitted intensity profile can be expressed by $\frac{1}{2}\cos^2(l\theta + \varphi_o + \alpha)$. For OV beams with same TCs and opposite signs, the transmitted intensity values are maximum at the azimuth angles $\theta_{\max} = \frac{n\pi - \varphi_o - \alpha}{|\ell|}$ $(n = 0,1, \ldots, |2\ell| - 1)$, leading to $|2\ell|$ lobes.

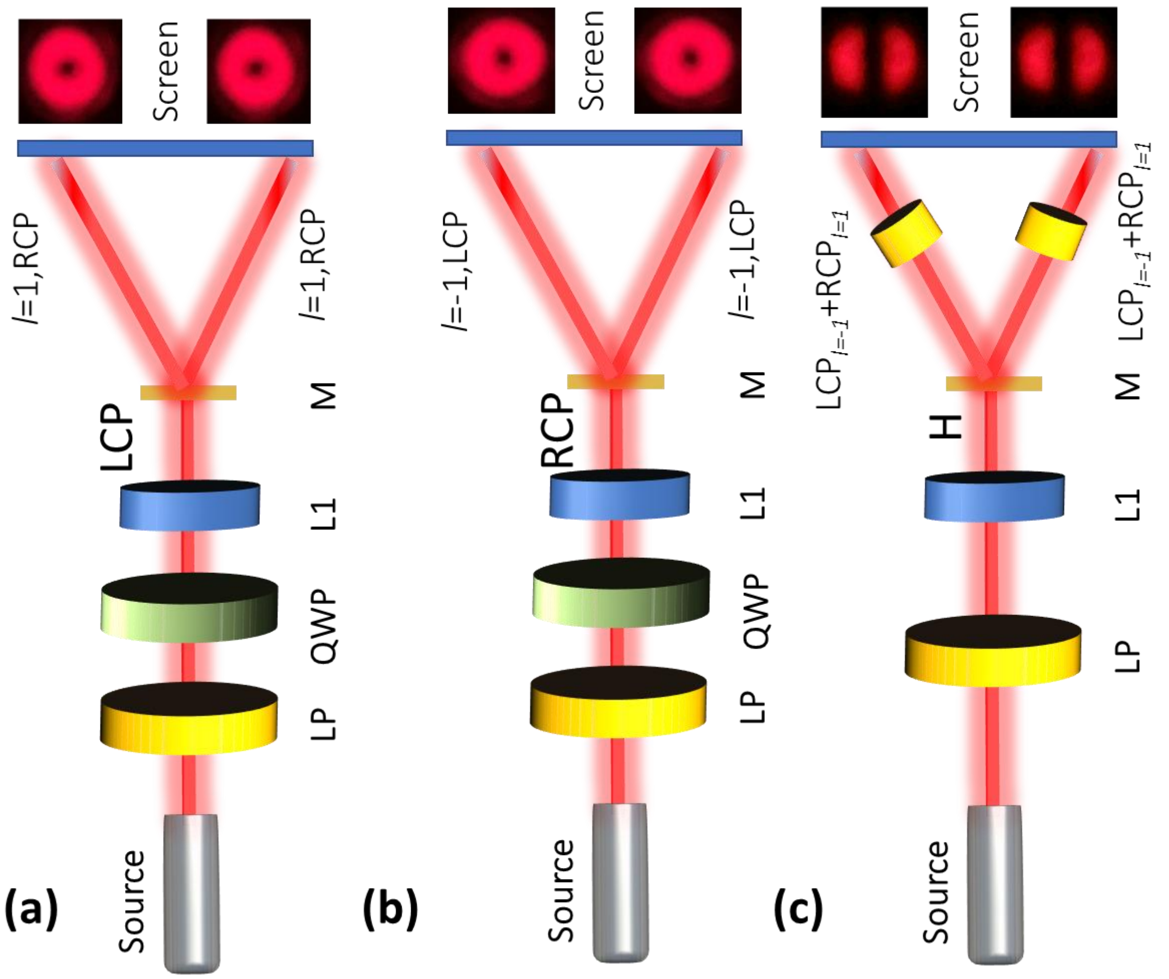

Figure S2: Sample Characterization. (a) Upon the illumination of a pure RCP light beam, the deflected light forms a pair of OVs at an angle of 12.5° with $l = 1$. (b) Upon the illumination of a pure LCP light beam, the deflected light forms a pair of OVs at an angle of 12.5° with $l = -1$. (c) When the input light is horizontally polarized (H), the resultant beam is the superposition of RCP and LCP OVs. The superposition is diagnosed by passing light through an analyzer. The direction of an analyzer is kept close to 0°. Insets show the transmitted intensity patterns captured at 650 nm.

Supplementary Section 4: Directional Edge imaging for Different USAF Chart Targets

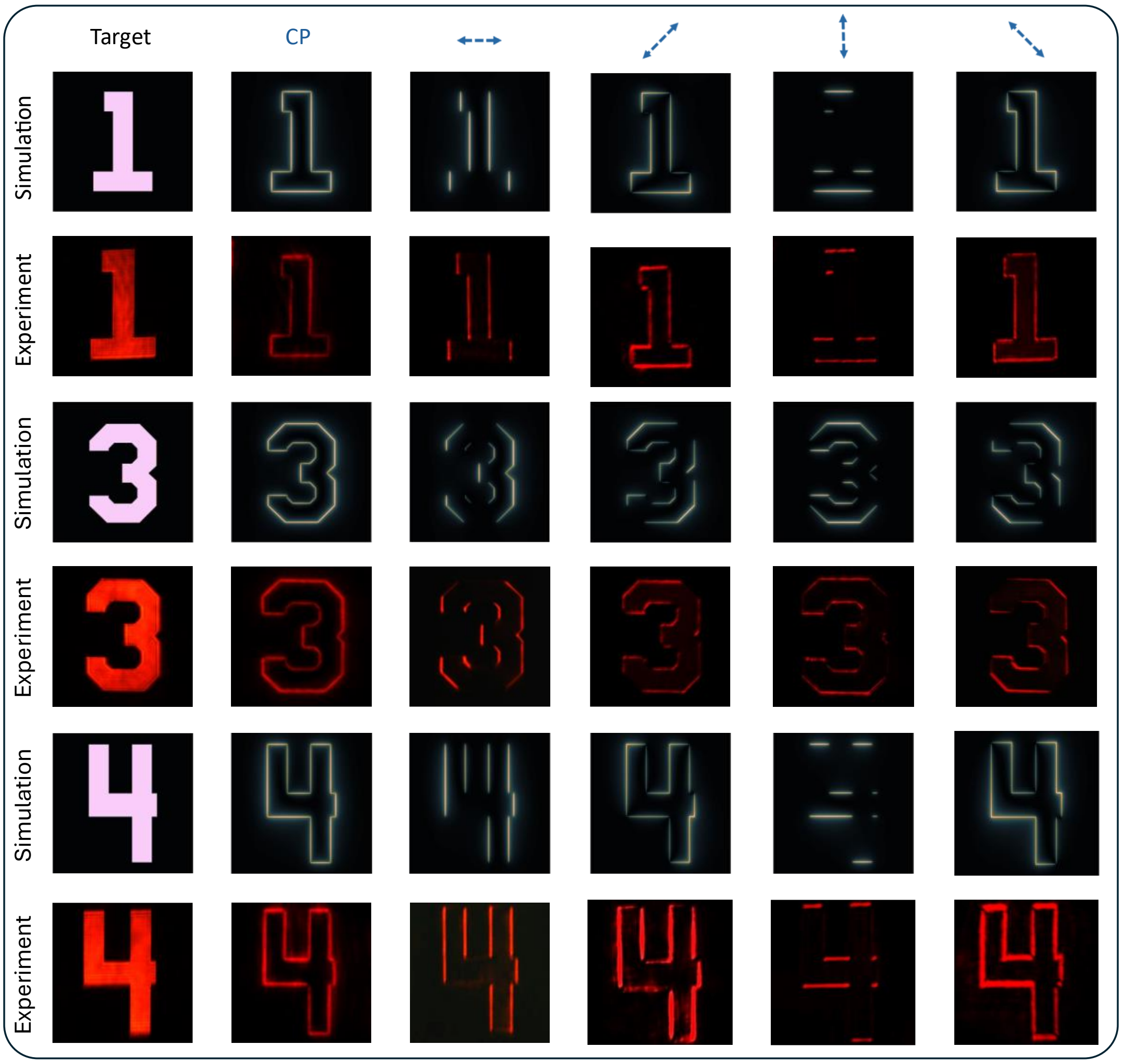

Figure S3: Directional edge imaging. The bright-field (1st column), conventional edge enhanced image (2nd column) and direction edge enhanced images (3rd- 6th column) of USAF resolution test target. A QWP is placed after a LP to generate various polarization states. Blue arrows represent the incident polarization states. CP denotes circular polarization.

Supplementary Section 5: Quantitative Analysis

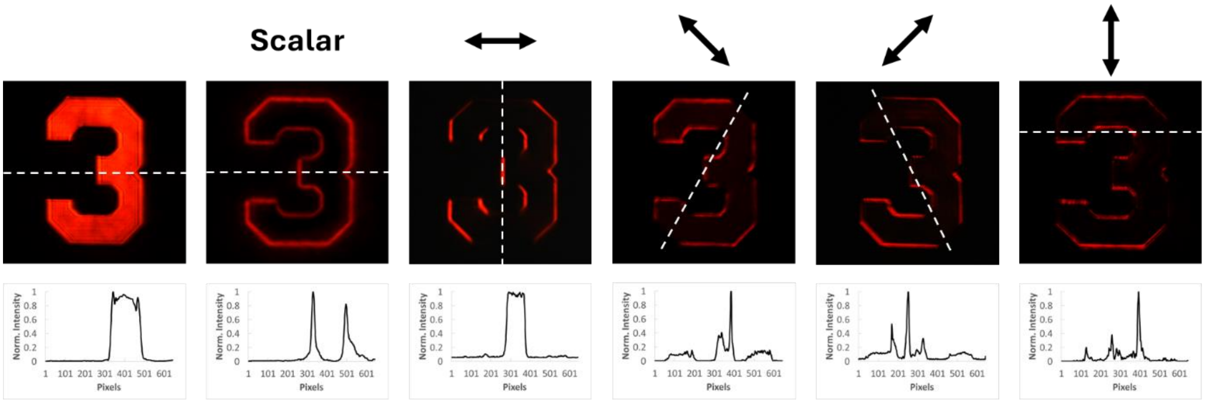

Figure S4. Intensity variation and edge sharpness. Edge profiles of bright field, scalar, horizontal polarization, 45°, 135°, vertical polarization. The bottom row shows intensity cross-sections taken along the dashed lines.

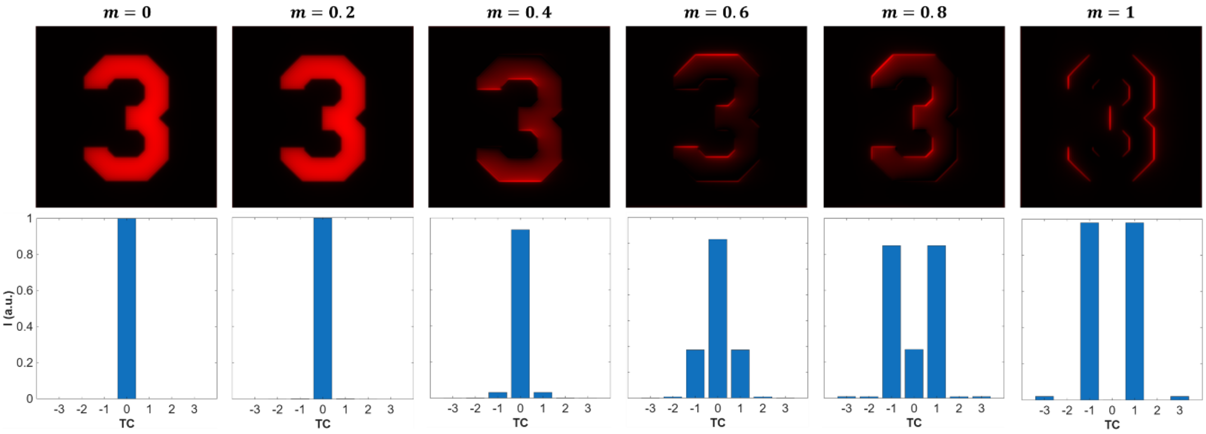

Figure S5. Effect of various polarization orders. The top row shows the directional edge imaging under various polarization orders ranging from 0 to 1. Bottom row shows the corresponding OAM spectra

Supplementary Section 6: VVB Polarization Modification and Correction

VVB can be described as the superposition of two orthogonal circularly polarized scalar POVs with conjugated TCs[3]

$$\boldsymbol{E_{VVB}} = E_R e^{i(\varphi_o)}|\boldsymbol{R}\rangle + E_L e^{-i(\varphi_o)}|\boldsymbol{L}\rangle, \qquad (S2)$$

where

$$|\boldsymbol{R}\rangle = e^{im\psi}\begin{bmatrix}1\\-i\end{bmatrix},$$

and

$$|\boldsymbol{L}\rangle = e^{-im\psi}\begin{bmatrix}1\\ i\end{bmatrix}.$$

Where m is the polarization order, φ_o is the initial phase. $|\boldsymbol{R}\rangle$ and $|\boldsymbol{L}\rangle$ are the right- and left-handed components. E_R and E_L are the amplitudes of RCP and LCP, respectively. Jones calculus is usually employed to investigate the transmission process and polarization distribution. Therefore, the Jones vector for VVBs can be written as[4]

$$\boldsymbol{E} = \begin{bmatrix}\cos(m\psi + \varphi_o)\\ \sin(m\psi + \varphi_o)\end{bmatrix}. \quad (S3)$$

various arbitrary polarization order by manipulating m and φ. The polarization distributions can be revealed after passing light through an analyser, whose Jones matrix is given as[1]

$$\boldsymbol{J_{LP}} = \begin{bmatrix}\cos^2\alpha_A & cos\alpha_A sin\alpha_A\\ cos\alpha_A sin\alpha_A & sin^2\alpha_A\end{bmatrix}, \qquad (S6)$$

where, α_A is the angle between the transmission axis of analyser and x-axis. The resultant polarization state can be presented as

$$\boldsymbol{P} = \boldsymbol{J_{LP}}\boldsymbol{E_{VVB}} = \begin{bmatrix}\cos(m\psi + \varphi_o - \alpha_A)\cos(\alpha_A)\\ \cos(m\psi + \varphi_o - \alpha_A)\sin(\alpha_A)\end{bmatrix}. \quad (S7)$$

Above polarization state can be further modified with the help of HWP. The jones matrix for HWP can be expressed as

$$\boldsymbol{J_{HWP}} = \begin{bmatrix}cos2\alpha & sin2\alpha\\ sin2\alpha & -cos2\alpha\end{bmatrix}. \quad (S11)$$

The jones vector for modified VVB can be described as

$$\boldsymbol{E_{VVB_{new}}} = \boldsymbol{J_{HWP}}\boldsymbol{E_{VVB}} = \begin{bmatrix}cos(2\alpha - m_n\psi - \varphi_o)\\ sin(2\alpha - m_n\psi - \varphi_o)\end{bmatrix}, (S12)$$

The above equation shows that the outgoing beam is still a VVB, but the polarization order is opposite to that of an incident beam (**Figure S6**) and the polarization direction is rotated by 2α.

To address this limitation, we add another HWP

$$\boldsymbol{E}_{\boldsymbol{VVB}_{\boldsymbol{new}}} = \boldsymbol{J}_{\boldsymbol{HWP2}}\boldsymbol{J}_{\boldsymbol{HWP1}}\boldsymbol{E}_{\boldsymbol{VVB}} = \begin{bmatrix} cos(2(\alpha_2 - \alpha_1) + m\,\theta + \varphi_o) \\ sin(2(\alpha_2 - \alpha_1) + m\,\theta + \varphi_o) \end{bmatrix}$$

The resultant polarization state is

$$\boldsymbol{P}_{\boldsymbol{n}_{\boldsymbol{new}}} = \boldsymbol{J}_{\boldsymbol{LP}}\boldsymbol{E}_{\boldsymbol{VVB}_{\boldsymbol{new}}} = \begin{bmatrix} \cos\left(\alpha_A + 2(\alpha_2 - \alpha_1) + m_n\psi + \varphi_o\right)\cos(\alpha_A) \\ \cos\left(\alpha_A + 2(\alpha_2 - \alpha_1) + m_n\psi + \varphi_o\right)\sin(\alpha_A) \end{bmatrix}. \quad (S13)$$

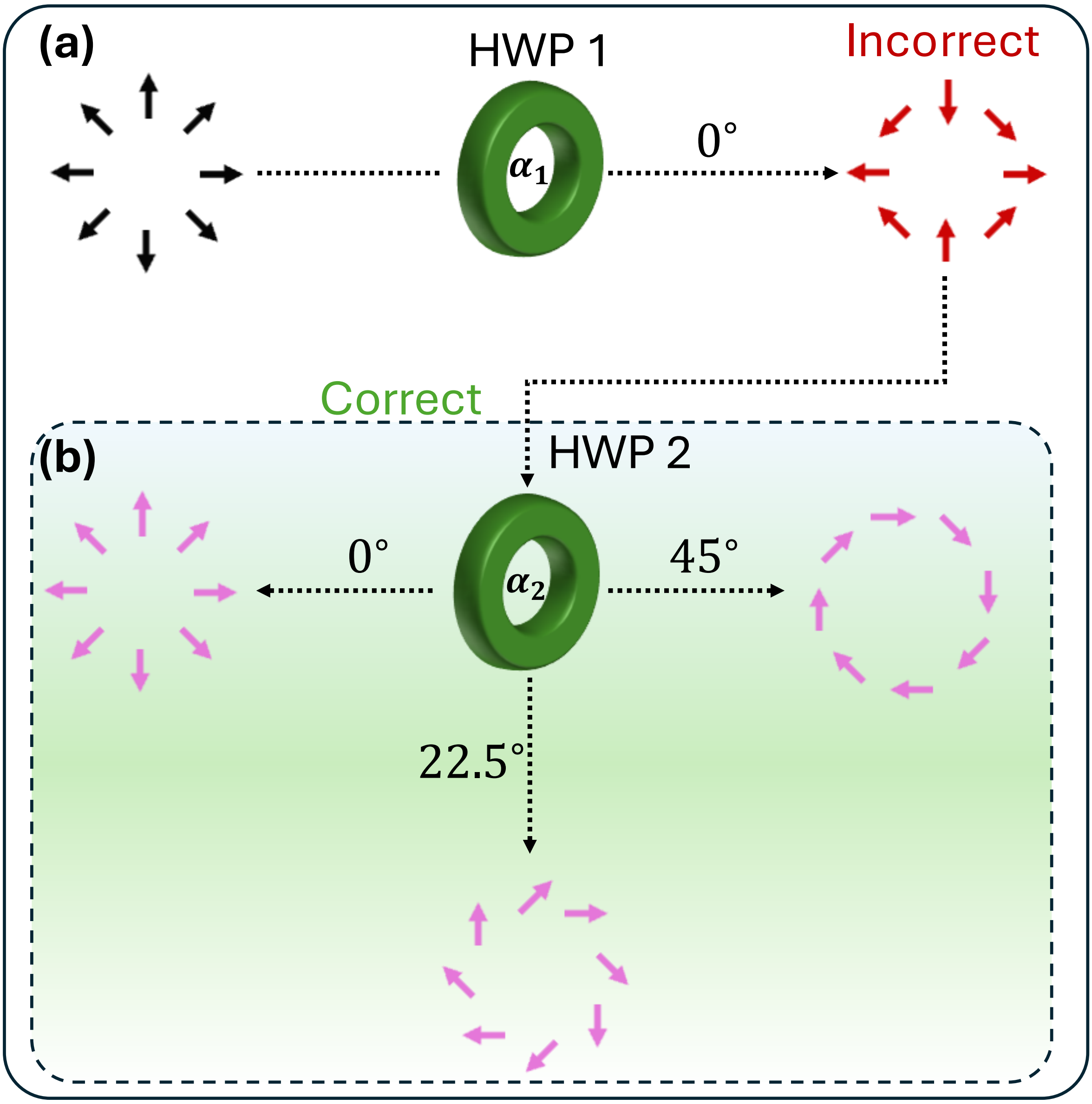

Figure S6: Polarization Correction. (a) Radial polarization modification using a single HWP1. Black arrows indicate the original polarization, and red arrows show the resulting modified but incorrect polarization profile. (b) Correct polarization profile achieved after the beam passes through the HWP2. Pink arrows represent the new polarization orientations at different values of α_2.

Supplementary Section 7: Directional Edge Imaging of Ipomoea Root and Onion Epidermal Cell

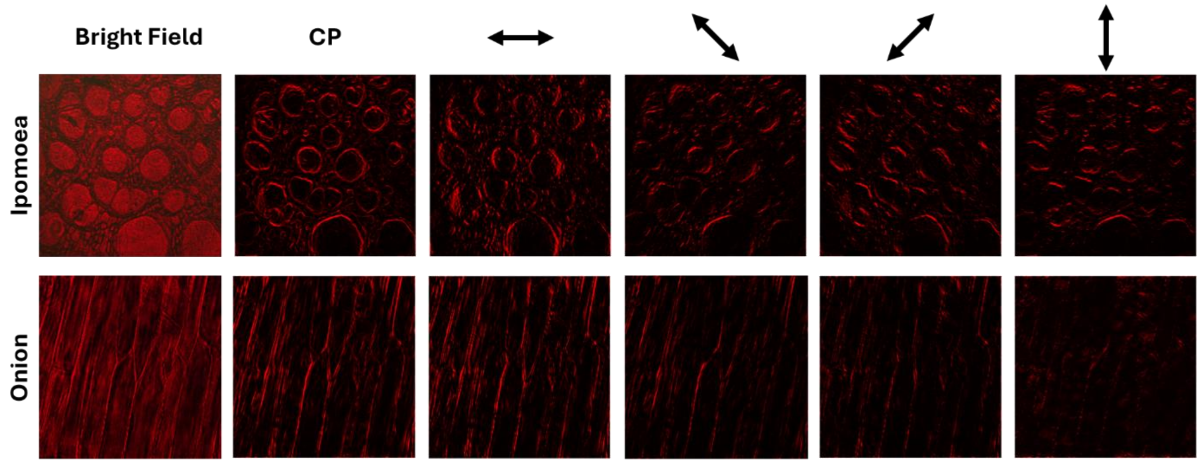

Figure S7. Directional edge imaging of biological samples. Measured intensity profiles of (a) Ipomoea root and (b) onion epidermal cells.

Supplementary Section 8: Field of View (FOV) Measurement

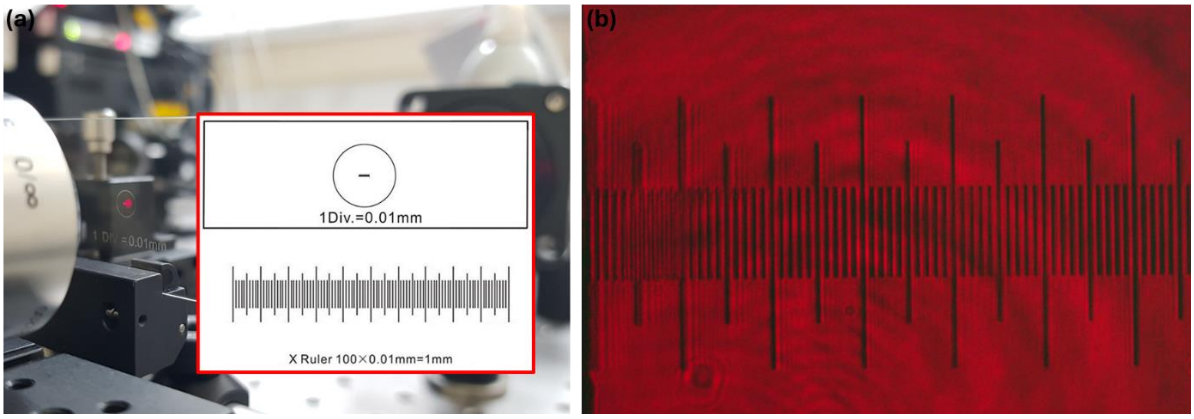

Figure S8. FOV measurement. (a) Target calibration scale (b) Microscopy image of scale.

Supplementary Section 9: Motorized Control

Supplementary Movie 1: Motorized Rotation of the Analyzer for Automated Polarization Tuning

References

1. Yue F, Wen D, Zhang C, Gerardot BD, Wang W, Zhang S*, et al.* Multichannel polarization-controllable superpositions of orbital angular momentum states. *Adv Mater* 2017, **29**(15)**:** 1603838.

2. Ma A, Intaravanne Y, Han J, Wang R, Chen X. Polarization Detection Using Light's Orbital Angular Momentum. *Advanced Optical Materials* 2020, **8**(18)**:** 2000484.

3. Maurer C, Jesacher A, Fürhapter S, Bernet S, Ritsch-Marte M. Tailoring of arbitrary optical vector beams. *NJPh* 2007, **9**(3)**:** 78.

4. Li D, Chang C, Nie S, Feng S, Ma J, Yuan C. Generation of elliptic perfect optical vortex and elliptic perfect vector beam by modulating the dynamic and geometric phase. *Applied Physics Letters* 2018, **113**(12)**:** 121101.

5. Li P, Zhang Y, Liu S, Ma C, Han L, Cheng H*, et al.* Generation of perfect vectorial vortex beams. *Optics letters* 2016, **41**(10)**:** 2205-2208.
